# Evaluating EHR-Integrated Digital Technologies for Medication-Related Outcomes and Health Equity in Hospitalised Adults: A Scoping Review

**DOI:** 10.1007/s10916-024-02097-5

**Published:** 2024-08-23

**Authors:** Sreyon Murthi, Nataly Martini, Nazanin Falconer, Shane Scahill

**Affiliations:** 1 School of Pharmacy, Faculty of Medical & Health Sciences, University of Auckland, Auckland, New Zealand; 2 School of Pharmacy, University of Queensland, Brisbane, Australia

**Keywords:** Hospital, Medication management, Digital technology, Electronic health records, Patient safety, Health equity

## Abstract

The purpose of this scoping review is to identify and evaluate studies that examine the effectiveness and implementation strategies of Electronic Health Record (EHR)-integrated digital technologies aimed at improving medication-related outcomes and promoting health equity among hospitalised adults. Using the Consolidated Framework for Implementation Research (CFIR), the implementation methods and outcomes of the studies were evaluated, as was the assessment of methodological quality and risk of bias. Searches through Medline, Embase, Web of Science, and CINAHL Plus yielded 23 relevant studies from 1,232 abstracts, spanning 11 countries and from 2008 to 2022, with varied research designs. Integrated digital tools such as alert systems, clinical decision support systems, predictive analytics, risk assessment, and real-time screening and surveillance within EHRs demonstrated potential in reducing medication errors, adverse events, and inappropriate medication use, particularly in older patients. Challenges include alert fatigue, clinician acceptance, workflow integration, cost, data integrity, interoperability, and the potential for algorithmic bias, with a call for long-term and ongoing monitoring of patient safety and health equity outcomes. This review, guided by the CFIR framework, highlights the importance of designing health technology based on evidence and user-centred practices. Quality assessments identified eligibility and representativeness issues that affected the reliability and generalisability of the findings. This review also highlights a critical research gap on whether EHR-integrated digital tools can address or worsen health inequities among hospitalised patients. Recognising the growing role of Artificial Intelligence (AI) and Machine Learning (ML), this review calls for further research on its influence on medication management and health equity through integration of EHR and digital technology.

**Supplementary Information:**

The online version contains supplementary material available at 10.1007/s10916-024-02097-5.

## Introduction

The issue of medication harm in healthcare settings is a significant concern, contributing to considerable avoidable patient harm, increased healthcare costs, and mortality [1]. More than 50% of patient harm is preventable, with half of these cases directly related to medication use [2–4]. Medication harm contributes to a mortality rate of 0.3% among hospitalised patients [5]. Annually, global medication harm costs an estimated 42 billion USD, often due to problems such as inefficient medication management systems, staff shortages, and high workloads [1, 6, 7]. Effectively addressing these issues requires a multifaceted approach. This should include improving system processes, implementing advanced technology, completing comprehensive staff training, and promoting a safety culture within healthcare organisations, as advocated by the Institute of Medicine (IOM) and the World Health Organisation (WHO) [1, 4, 8].

Advances in digital technology hold significant promise in improving medication management through Electronic Health Records (EHRs) [9, 10], particularly through the Electronic Medication Management System (eMMS), which digitises the medication process [11]. In hospital settings, these processes involve prescribing, administering, monitoring, and reviewing medications [4]. The eMMS enables clinicians, such as doctors, nurses, and pharmacists, to manage these tasks electronically, thus eliminating paper documentation [11]. However, despite the success of EHRs in digitising clinical records, consolidating patient data, promoting efficient clinical decision-making, and streamlining workflows, medication harm still persists [12–19]. Factors that contribute to medication harm include alert fatigue due to irrelevant warnings, lack of real-time medication harm detection, and the absence of personalised clinical guidance for medication dosage and monitoring [12–19]. The key to reducing medication harm when using EHRs lies in prompt and effective identification, monitoring, and proactive responses to any harm that occurs [12–19].

To tackle the complex factors that contribute to medication harm, there is a critical need to integrate innovative digital technologies into EHR systems [12–19]This highlights the importance of gaining a deeper understanding of the design, implementation, and integration of EHR-digital technology in healthcare settings [12–19]. Recent research suggests that digital technologies such as clinical decision support systems (CDSS), predictive algorithms, and large data analysis software can mitigate medication harm and improve patient outcomes [20–23]. Through these tools, hospital clinicians can identify patients at risk for medication harm or those who are currently experiencing harm in a timely manner [24]. They can provide real-time support, monitor patients using high-risk medications, and report errors, resulting in more precise and efficient clinical interventions [24]. Seamless communication among healthcare providers through EHR-digital technology integration can improve medication management, particularly in patients with complex medical histories or multiple comorbidities [25]. Consequently, this integration can support evidence-based prescribing, and reduce medication risks [25]. Furthermore, Artificial Intelligence (AI) and Machine Learning (ML) systems can detect trends and patterns, enabling proactive measures instead of reactive responses to medication harm [24].

Despite the significant progress made in utilising digital technologies to reduce medication errors within EHRs, there is a lack of comprehensive evaluations of the effectiveness and implementation of these technologies across diverse hospital settings. While studies emphasise the potential of tools such as CDSS, predictive algorithms, and AI, they often overlook addressing the implementation challenges and limitations of real-world deployment. There is a noticeable gap in research regarding how technology adoption differs across different hospital environments, the contextual factors that influence their implementation, how it impacts clinical workflows, and how it contributes to reducing medication harm over time. Importantly, socioeconomic factors, cultural influences, and health determinants affecting technology success in resource-constrained settings are often not considered [26]. This highlights the need for studies that evaluate the effectiveness of EHR-digital technology and the contextual factors that influence their implementation.

Understanding the implementation and integration of EHR-digital technology in medication management is essential for several reasons [24, 25, 27]. This approach supports the development of more effective and efficient eMMS, promoting patient-centred care by allowing more accurate and timely access to patient information for health professionals [24, 25, 27]. This approach also improves clinical decision making and helps shape future health policies by providing information on the benefits and challenges of integrating digital technologies with EHRs, which in turn provides a better framework to address medication harm [24, 25, 27].

Integrating a health equity lens during the development of digital health technologies is crucial to advance equitable innovation [28–30]. These approaches require the inclusion of health and social determinants at each stage of design, development, pilot testing, integration, implementation, and stakeholder engagement [28–30]. By addressing these determinants, it is possible to develop digital health solutions that actively enhance equity for disadvantaged groups [28–30]. On the contrary, digital technologies developed without these considerations, especially when created using historical data, missing data, or poor sample sizes, risk perpetuating health inequities, further excluding disadvantaged patient groups that already experience poor health outcomes [31–34].

## Purpose

The purpose of this scoping review is to identify and evaluate studies that examine the effectiveness and implementation strategies of Electronic Health Record (EHR)-integrated digital technologies aimed at improving medication-related outcomes and promoting health equity among hospitalised adults. Specially, we focus on:

- Population (P): Hospitalised adults
- Exposure (E): Digital technologies integrated with EHRs used by clinicians
- Outcome (O): Improvements in medication-related outcomes, such as adverse drug event (ADE) rates, and medication errors driven by EHR-digital technologies. Additionally, health equity outcomes, defined as changes in medication-related outcomes due to EHR-digital technologies for disadvantaged patient groups experiencing poor health outcomes.

This review was guided by two objectives:

- To appraise studies that evaluate the effectiveness of EHR-digital technology examining medication-related and health equity outcomes.
- To identify and analyse the factors that influence the implementation of EHR-digital technology within complex hospital systems.

## Methods

We adopted the Joanna Briggs Institute (JBI) guidelines for scoping reviews and report according to the Preferred Reporting Items for Systematic Reviews and Meta-Analyses extension for Scoping Reviews (PRISMA-ScR) [35, 36].

### Search Strategy

The search strategy was developed with research librarians and began with a preliminary search on Medline. After scanning the titles, abstracts, and keywords, the final search strategy conducted in the month of January 2023, was tailored to the following databases: Medline, Embase, Web of Science, and CINAHL Plus. The search identified primary peer-reviewed studies published in English from the inception of the database to January 2023.

Keywords and Medical Subject Headings (MeSH) used in the search included 'medication harm', 'electronic health records' and terms related to 'risk, detection, digital surveillance, and health inequities.' Furthermore, search terms focused on health equity for Indigenous and disadvantaged groups, such as 'Indigenous' or 'equit*' or 'inequit*' or 'disparity' or 'disparit*' or 'inequalit*' or 'Maori' or 'Maaori' or 'Pacific*' or 'Pasifika' or 'Pasefika'. The reference lists of identified studies were reviewed for additional sources. The literature search strategy is included in the Supplementary Materials section.

### Eligibility Criteria

Eligible studies for this review involved integrated digital technologies with EHR and aimed at improving medication management for hospitalised adults aged 18 years and older. Paediatric populations were excluded due to their unique requirements of varying weight-based medication dosing, prescribing, administering, and monitoring processes.

The studies needed to demonstrate the application of appropriate technologies in various aspects of medication management in hospital settings, including but not limited to real-time prediction, detection, monitoring, surveillance, management, guidance, and alert generation to aid clinicians such as doctors, nurses and pharmacists.

The primary outcomes of interest were medications-related outcomes, such as ADE rates and incidence, medication errors, and alert effectiveness, and the prevalence of alert fatigue. Additionally, we evaluated the impact of EHR-integrated digital technologies on stakeholders and implications on health equity. Health equity outcomes were defined as changes in medication-related outcomes due to EHR-digital technologies for disadvantaged patient groups experiencing poor health outcomes. We actively searched for studies that evaluated health equity outcomes in medication management driven by EHR-digital technologies. Furthermore, studies had to be available as primary, peer-reviewed, full-text literature published in English.

Excluded from this review were studies that relied on manual or paper-based methods to detect medication harm, those that used paper medication charts and those that focused exclusively on built-in functionalities of commercial EHR systems. Studies conducted in outpatient settings were also excluded, as were any review-type articles. Studies focusing on developing, implementing, evaluating, or comparing different models or algorithms without direct application to real-world clinical settings and those solely using historical datasets for analysis were not considered.

### Study Selection and Data Extraction Process

**Fig. 1:**
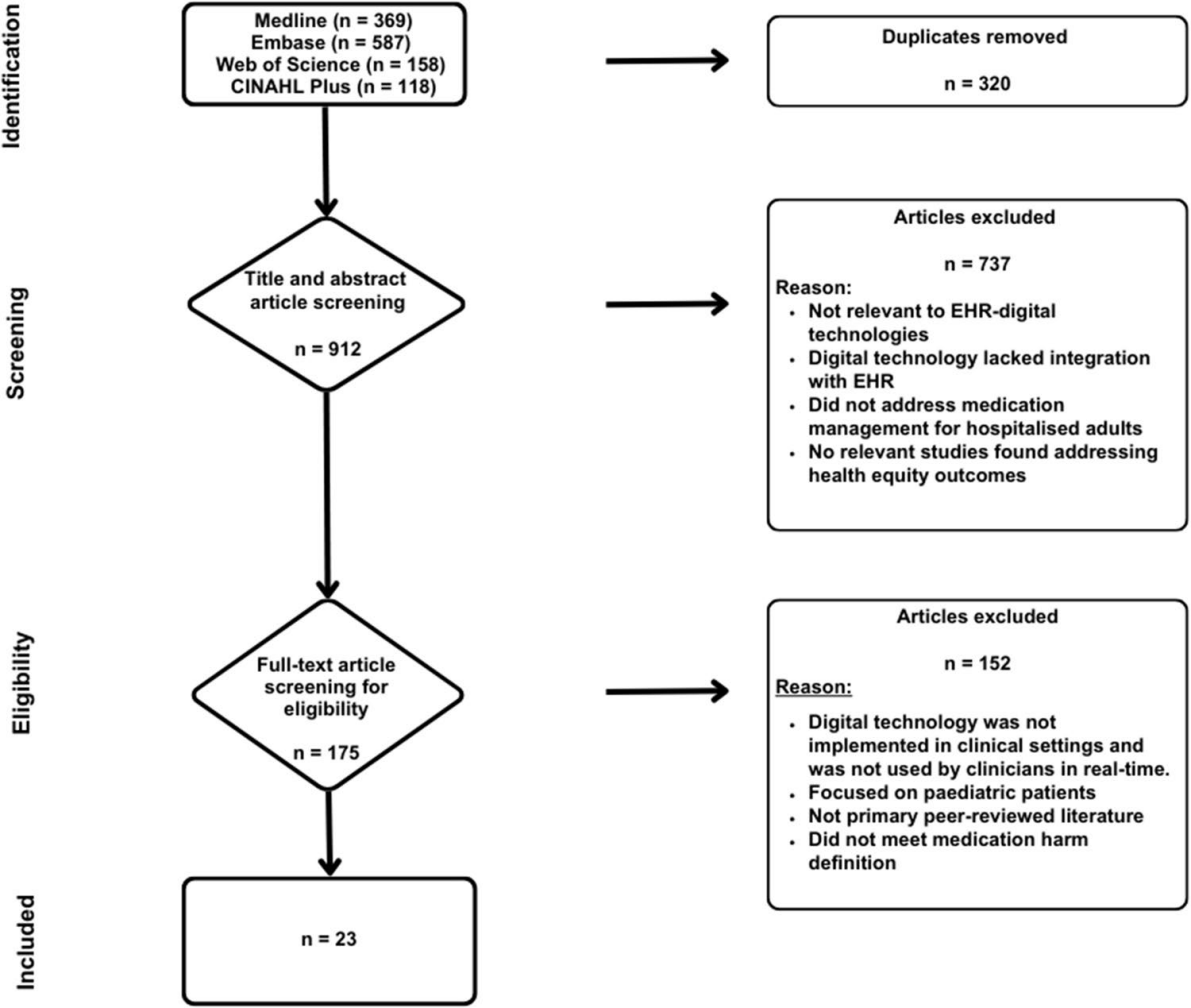
PRISMA flowchart for study selection

The title and abstract screening were performed by (SM), while the full-text review was done collaboratively by the research group (SM, NF, NM, SC), as reported in Fig. 1. Any ambiguities during the screening process were resolved through consensus discussion in regular research meetings. This review paid particular attention to the implications for health equity, assessing how digital technologies integrated with EHRs address or potentially exacerbate health inequities among hospitalised adults.

For data extraction and analysis, a structured data collection form was developed and tested, which included the following categories: author, year of publication, country, type of study design, functionality of the digital tool, method used and outcome measures. The implementation methods of the studies were evaluated using the Consolidated Framework for Implementation Research (CFIR) to understand contextual factors in complex healthcare settings [37–39]. Although this framework is used most frequently to measure and report implementation outcomes for complex interventions in primary studies, it was deemed valuable for this review to ensure that key learnings were described. Methodological quality and risk of bias were also evaluated using the NHLBI Quality Assessment Tool for Before-After (pre-post) studies with no control groups [40].

## Results

### Study Selection

In total, 1,232 studies were identified. After removing duplicates, 912 studies were screened by title and abstract, resulting in 175 studies being included in the full text review. A total of 23 studies met the inclusion criteria and were included in this scoping review. No relevant studies were found using health equity search terms, specifically addressing health equity outcomes in the context of medication management driven by EHR-digital technologies used by clinicians for hospitalised adults. Moreover, the 23 included studies did not provide any data on health equity outcomes.

### Study Characteristics

Table 1 provides an overview of the included studies, demonstrating the use of a variety of study designs, some of which used mixed methods. These included retrospective analyses (*n* = 7) [41–47], prospective evaluations (*n* = 6) [48–53], pilot interventions (*n* = 2) [54, 55], descriptive studies (*n* = 2) [56, 57], quasi-experimental designs (*n* = 1) [58], pre and post-implementation studies (*n* = 1) [59], and mixed methods (*n* = 4) [60–63]. The most common designs were retrospective [41–47, 60–63] and prospective [48–52]. From 2008 to 2022, these studies were carried out in 11 countries, including Saudi Arabia, Finland, the United States, Spain, France, Korea, Sweden, Israel, Switzerland, Germany and the Netherlands (45–67). Notably, the USA contributed the most studies, a total of nine (47–49,52,53,55,60,61,67).

**Table 1:** Study characteristics

#	Author	Year of publication	Country	Study design type	Digital tool functionality	Method used	Medication-related outcome measure	Health equity outcomes
1	Al-Jazairi et al. [41]	2019	Saudi Arabia	Retrospective	• Detects medication doses outside recommended range	• Retrospective analysis of dose range alerts	• Total rate of overridden alerts • Percentage of accepted alerts	• Not reported
2	Böttiger et al. [54]	2018	Finland	Pilot, observational	• Provides risk profiles for adverse events associated with multiple medications	• Approximately 1400 substances were scored for risk assessment • Algorithms developed for individual risk profiles	• Risk profile of adverse events associated with multiple medicines • Data from pilot for functionality and acceptance of the tool	• Not reported
3	Chernoby et al. [42]	2020	USA	Retrospective quasi-experimental, multicentre	• Alerts high-risk QTc-prolonging medication-related adverse events	• Evaluation of provider response to alerts	• Proportion of prescriptions triggering high risk QTc-prolonging alerts continued without intervention	• Not reported
4	Daniel et al. [43]	2021	USA	Retrospective, multicentre	• Real-time alerts on oral anticoagulation adverse drug events	• Analysis of clinical data from EHR	• Reduction in coded oral anticoagulant adverse drug events • Prompts for pharmacist intervention	• Not reported
5	Drago et al. [59]	2020	USA	Pre- and post-implementation	• Aids in reducing potentially inappropriate medications	• Pre- and post-intervention study analysis	• Percentage of orders consistent with the recommendations for geriatric dose, average dose, and total daily dose in the 12 months before and after implementation	• Not reported
6	Ferrandez et al. [44]	2016	Spain	Retrospective, observational	• Computerised warning system • Monitors, identifies, and alerts potential drug-related problems	• Review alerts generated	• Proportion of alerts generated and required pharmacist intervention	• Not reported
7	Hackl et al. [58]	2013	France	Quasi-experimental	• Detects and alerts adverse drug events	• Controlled time series analysis of adverse event data in three departments	• Monthly rates of possible adverse drug events	• Not reported
8	Hirsch et al. [56]	2021	USA	Descriptive	• Provides dose and frequency recommendations based on indication and kidney function	• Design, development and deployment of automated dose and frequency recommendations	• Acceptance rate of automated recommendations	• Not reported
9	Jha et al. [48]	2008	USA	Prospective	• Detects and alerts adverse drug events or potential adverse drug events	• Screening and identification of potential drug-drug interactions	• Identification of potential drug‒drug interactions Evaluate clinical relevance of identified potential drug‒drug interactions	• Not reported
10	Kang et al. [45]	2018	Korea	Retrospective case‒control	• Predicts and alerts risk of medication errors	• 4 phases: (1) development of a risk scoring algorithm, (2) validation of the algorithm, (3) development of the digital technology in the EHR system, and (4) evaluation of the digital technology after clinical application	• Predictive validity of medication errors	• Not reported
11	Lim et al. [49]	2016	USA	Prospective	• Detects and alerts adverse drug events	• Evaluation of trigger detection messages using the Institute for Healthcare Improvement (IHI) guidelines	• Positive predictive value (PPV) of triggers	• Not reported
12	Mannheimer et al. [57]	2008	Sweden	Descriptive	• Screens for potential drug‒drug interactions	• Identification of potential drug‒drug interactions	• Evaluation of clinical relevance of identified potential drug‒drug interactions	• Not reported
13	Naor et al. [63]	2022	Israel	Retrospective and prospective	• Detects, screens, identifies, and alerts drug‒drug interactions and potentially inappropriate medications	• Deep learning Artificial Intelligence (AI) model to identify and intercept potential medication-related real-time risks	• Identification of medication errors and adverse events	• Not reported
14	Niedrig et al. [60]	2016	Switzerland	Retrospective, pre- and post-implementation	• Screens paracetamol overdose and issues alerts	• Identify patients at risk of repeated paracetamol overdose (≥ 5 g/day for ≥ 3 subsequent days)	• Quantify relevant paracetamol overdosing alert with high specificity	• Not reported
15	Niedrig et al. [61]	2016	Switzerland	Retrospective	• Screens metformin overdose with renal impairment and issues alerts	• Detect metformin prescriptions and automatically check the estimated glomerular filtration rate (eGFR) of patients using the Chronic Kidney Disease Epidemiology Collaboration formula	• Number of automated alerts generated by the algorithm • The number of patients for whom recommendations on metformin therapy were issued • Prescribers' compliance with these recommendations	• Not reported
16	Peterson et al. [55]	2014	USA	Pilot intervention	• Identifies and alerts for potentially inappropriate medications or high anticholinergic scores • Displays cumulative administration of narcotics and benzodiazepines over a 48-h period	• Electronic PIMS dashboard for medication review • Clinical pharmacist intervention for physician recommendations	• The proportion of individuals identified by the electronic PIM dashboard • The number of participant-medication pairs that require manual review • The number of inappropriate medication orders that warrant intervention • Clinician response to pharmacist recommendations	• Not reported
17	Pouliot et al. [46]	2018	USA	Retrospective	• Checks for inappropriate medication administration during epidural therapy	• Retrospective cohort chart review	• Inappropriate medication administration determined by the American Society of Regional Anaesthesia Guidelines on Regional Anaesthesia in the Patient Receiving Antithrombotic or Thrombolytic Therapy Proportion of enoxaparin use	• Not reported
18	Rommers et al. [62]	2011	Netherlands	Retrospective and prospective	• Detects and alerts potential adverse drug events	• Conventional medication surveillance system with retrospective checks by hospital pharmacists • Computerised adverse drug event alerting system using clinical rules	• The total number of alerts, the number of unique alerts, number of patients with alerts, and number of interventions by the hospital pharmacist generated by the two systems	• Not reported
19	Roten et al. [50]	2010	Switzerland	Prospective, observational, and comparative	• Identifies patients at risk of drug-related problems	• Developing electronic queries based on literature review, clinical pharmacists’ experience, and programming feasibility	• Sensitivity and specificity of electronic queries developed to identify inpatients at risk of drug-related problems (DRP)	• Not reported
20	Segal et al. [51]	2019	Israel	Prospective	• Detects and alerts medication errors	• Evaluate the accuracy, validity, and clinical usefulness of medication error alerts generated by machine-learning algorithms to identify and intercept potential medication prescription errors in real time	• Alert burden • Alert accuracy and clinical relevance • Physician's response to the alerts	• Not reported
21	Seidling et al. [52]	2010	Germany	Prospective, open, monocentric study with two sequential phases	• Triggers warning when doses exceed the individualised maximum recommended therapeutic dose by 30%	• Development, implementation, and review of an algorithm-based clinical decision support system (CDSS) determining individualised upper dose limits	• Frequency of excessive doses before and after intervention • Evaluate the impact of reducing prescription rate of excessive doses compared to baseline	• Not reported
22	Skalafouris et al. [53]	2022	Switzerland	Prospective observational	• Screens and detects twenty high-risk adverse drug events	• A clinical decision support system (CDSS) constructed as a clinical, rules-based system using facts and IF–THEN rules to screen high-risk situations in EHR	• The intervention's positive predictive value (PPV) to assess the performance in detecting high-risk situations and its impact on clinical pharmacists' activities • Clinical PPV with and without pharmacist intervention	• Not reported
23	Waitman et al. [47]	2011	USA	Retrospective observational	• Identifies, alerts, and displays on dashboard risk of adverse drug events for high-alert medications	• Real-time web-based surveillance application to organise patient data into dashboards based on provider-entered orders for high-alert medications	• Proportion of cases with a positive alert relative to the number of cases exposed to the drug	• Not reported

The primary objectives of these studies were to evaluate the clinical validity of digital technologies that check dose ranges [41], develop risk profiles for adverse events associated with polypharmacy [44], and assess the impact of digital technologies targeting specific drug-related issues, such as QT interval-prolonging medications [42] and oral anticoagulant adverse drug events (ADE) [43]. Other objectives included designing digital technologies for the rapid detection of drug-related problems (DRP) [56], investigating the acceptance of ADE alerts [58], real-time surveillance dashboards aimed at early detection of ADE [47], and improving prescribing practices for hospitalised older adults [59]. The duration of these studies varied considerably, ranging from short-term analyses of days to weeks [41, 55] to longer-term evaluations spanning several months [42–54, 62, 63] and even years [56–61].

### EHR-Digital Medication Management Technology and Their Functionalities

The reviewed studies investigated various digital technologies integrated with EHRs for the management of medications in hospitals. These technologies included screening tools [50, 57, 63], CDSS [41, 42, 52–54, 54, 58, 59], real-time surveillance [43, 47–49], predictive analytics models [45, 51], automated alert algorithms [44, 60, 61], reminders, and recommendations based on evidence-based guidelines and patient-specific data [46, 55, 56, 62]. Overall, these digital tools demonstrated various functionalities to improve medication management. For example, one study reported the use of digital technology to detect doses of medications that exceed the recommended range [41]. Another study developed capabilities to provide polypharmacy related adverse event risk profiles using a scoring system for approximately 1400 medications [54]. Two studies developed alert systems for high-risk medications such as QTc-prolonging medications and oral anticoagulation, respectively [42, 43]. In a different approach, a computerised warning system was developed to monitor, identify, and alert healthcare providers of potential drug-related problems [44]. Other studies focused on dosage and frequency recommendations. One study used EHR-digital technology integration to generate recommendations based on the patient’s specific indication and kidney function [56], while another used the integration to reduce potentially inappropriate medications in older adults [59].

### Outcome Measure

A variety of metrics were used to evaluate the outcomes of EHR-digital integration in medication management. Key metrics included the total number and percentage of alerts that were overridden and accepted [41], the proportion of high-risk medication prescriptions that were continued without intervention [42], and the acceptance rates of automated recommendations tailored to the indication of the medication and the patient's kidney function [56]. Studies also determined the positive predictive value (PPV) of triggers for identifying ADE and the predictive validity of medication errors [45, 49, 53]. In addition, the clinical relevance of identified drug‒drug interactions and the specificity of alerts in situations such as overdosing were measured [48, 60, 61]. The influence of EHR-digital technology on prescribing practices was evaluated by examining the percentage of orders that adhered to geriatric dose recommendations and the proportion of patients flagged for potentially inappropriate medications (PIMs) [55, 59]. Furthermore, one study examined the alert burden, accuracy, and clinical relevance of machine learning algorithms in detecting medication errors while also analysing physicians' responses to these alerts [51].

## Key Findings from Studies

The studies included in this review provide insights into the success and challenges of using EHR-based digital technology to improve medication management. These studies aimed to evaluate the effectiveness of various tools designed for hospital clinicians, including alert systems, screening and surveillance tools, risk assessment systems, electronic trigger tools, dashboards, and intervention tools.

### Effectiveness and Challenges of Alert Systems

Several studies have analysed the effectiveness and challenges of alert systems in hospitals. A particular study reported a 96% acceptance rate of automated recommendations by healthcare professionals, indicating a strong trust and utility of these digital prompts [56]. Another study generated 516 high-priority alerts, of which 23% were related to actual ADEs and 15% were related to potential ADEs [48]. This demonstrates the system's ability to identify medication safety issues [48].

A study investigating a CDSS found that alerts were generated for only 0.4% of medication orders, indicating a low alert burden [51]. However, 60% of these alerts were triggered after the medication was administered due to changes in the patient's condition [51]. Importantly, 85% of these alerts were clinically valid and 80% clinically useful, influencing changes in 43% of subsequent prescription orders [51].

The efficacy of automated alert systems varies, as one study reported that 72% of the alerts generated were clinically relevant during the prospective phase [63]. Over a 3-year period, another study found 2,145 automated alerts generated, with a 79% compliance rate leading to a reduction in metformin overdose incidents from 64 to 47 [61]. Despite this success, the system was unable to prevent eight patients from having metformin-associated lactic acidosis, probably due to rapid deterioration of renal function [61]. Focusing on paracetamol overdoses, the alerts of one study resulted in prescription changes in 21 out of 23 patients (91.3%), with eight alerts related to conditions such as cachexia or alcoholism resulting in six adjusted prescriptions [60]. In particular, no cases of overdose exceeding 5 g/day for more than 2 days after implementation were reported [60].

In a separate study, the implementation of a CDSS that provided recommendations and warnings led to a reduction in the incidence of inappropriate medication administration after epidural anaesthesia (from 12.8% to 6.3%), although these decreases were not statistically significant [46]. However, a significant reduction in the incidence of inappropriate administration of enoxaparin was observed after epidural anaesthesia, dropping from 3.9% to 0% after implementation [46]. Additionally, a pharmacy warning system was able to detect 2,808 potential DRPs, 20% of which required pharmacist intervention. These interventions were primarily related to dosage and laboratory tests, particularly for medications used to treat infections, pain, and heart conditions [45].

One study reported that a new system produced fewer, but more accurate, alerts than traditional methods [62]. Furthermore, the adoption of a CDSS was associated with a significant 20% reduction in overprescribing [52]. In another study, the effectiveness of digital alert systems in predicting medication errors was found, with values of the area under the curve (AUC) exceeding 0.8, indicating strong predictive accuracy [45]. In contrast, during a 10-day trial another study found that 95% of dose range checking alerts were clinically invalid, and prescribers ignored 96% of these alerts [41]. This study also identified issues associated with prescribed dose inaccuracies, with 33% involving overdoses and 52% involving underdoses, highlighting significant challenges in ensuring alert usability and accuracy of prescribed doses [41].

### Screening and Surveillance Tool

The implementation of electronic screening and surveillance tools plays an essential role in the detection of high-risk situations and patients, thus improving the early detection of potential medication-related problems. [47, 53]. An example of such a tool is a screening tool that is integrated with pharmacist-operated dashboards, which improved the detection rates and clinical positive predictive value (PPV) of alerts [53]. The overall intervention PPV of this tool was 20.1%, with variations between categories: 26.9% for abnormal laboratory value alerts, 3.1% for contraindicated medications, 28.2% for drug‒drug interaction alerts, and 14.3% for inappropriate route of administration alerts [53]. Furthermore, when pharmacists participated in filtering alerts, there was a significant increase in clinical PPV of 71.0% compared to 14% without intervention [53]. Another electronic dashboard for pharmacists reviewing medications for elderly patients reported a 78% acceptance rate for clinical recommendations [55]. These recommendations focused mainly on discontinuing inappropriate medication, adjusting the dose or frequency, substituting medication, and improving clinical monitoring [55].

Various studies have reported improvements in the management of specific drug-related problems (DRP) due to the use of electronic screening and surveillance tools. For example, a study highlighted a significant reduction in the continuation of orders after QTc alerts, from 81.6% to 37%, indicating enhanced responsiveness to potential cardiac risks, with a p-value of 0.02 [42]. Real-time surveillance tools were also associated with a significant decrease in the incidence of oral anticoagulant ADE, from 69 to 41% (p < 0.001), particularly in patients receiving a single oral anticoagulant [43]. In older adult care, a study reported an increased adherence to recommended geriatric doses for the ten most prescribed medications, with nine of the ten showing a statistically significant increase in the uptake of recommended doses (p-value < 0.05) [59]. This indicates that improved adherence to dosage guidelines contributes to safer medication practices among the elderly [59]. There was also a trend towards prescribing lower average and total daily doses for most medications after implementation, suggesting a more cautious approach [59].

Furthermore, a screening tool demonstrated 85.1% sensitivity and 60.4% specificity to detect DRPs [50]. It was found that of the 150 potential drug-drug interaction (DDI) pairs identified during an investigation, only 24 were considered clinically relevant [57]. The study highlighted significant potential pairs of DDI involving angiotensin-converting enzyme (ACE) inhibitors and diuretics, emphasising the importance of monitoring these potentially harmful interactions to enhance patient safety [57].

### Risk Assessment System and Electronic Trigger Tool

A pharmacological risk assessment system received positive user feedback and successfully identified risks associated with 732 drugs in 933 uses, with the most common risks being constipation, sedation, and bleeding [54]. Moreover, an electronic trigger tool aimed at enhancing ADE detection identified 27 ADEs, however, a low PPV of 0.31 was reported. This was attributed primarily to false positives, especially in cases involving the drug phytonadione in specialised patient populations such as those with cardiogenic shock and haematologic complications [49]. From 2007 to 2012, another decision support tool identified 3,586 ADE, successfully detecting 24 of 27 ADE types [58]. Despite these achievements, the implementation of the tool did not significantly reduce the total number of ADEs [58]. Although healthcare professionals acknowledged the utility of the tool, there was no definitive evidence to suggest its effectiveness in markedly reducing ADE [58].

## Consolidated Framework for Implementation Research (CFIR)

The CFIR framework was used to assess the factors that influence the implementation of EHR-digital technology in complex hospital systems [39]. Table 2 summarises these findings. A more detailed analysis is provided in the Supplementary Materials.

**Table 2:** Summary of the CFIR analysis [39]

CFIR Main Domain	Subdomain	Observations from Studies
Intervention	Innovation Source	Almost all studies adhered to this subdomain, indicating a strong innovation source across interventions [41–46, 48–51, 54–60, 62, 63].
	Evidence-based	The domain was addressed inconsistently, with only a few studies showing evidence-based approaches [46, 48, 51, 54, 55, 60, 63].
	Relative Advantage	Most studies demonstrated the relative advantage of their digital technologies [41–45, 49, 51, 54–60, 63].
	Adaptability	Studies have shown that interventions are adaptable to various settings.
	Trialability and Complexity	Commonly addressed, reflecting the practical and intricate nature of EHR-digital technology integration.
	Design	Studies often place less emphasis on design aspects, suggesting a potential gap in research in this area.
	Cost	The financial aspects of the integration of EHR and digital technology were not addressed in the studies.
Outer Setting	Overall	External factors, such as policies and laws, were under-represented, indicating a lack of focus on them.
Inner Setting	Information Technology Infrastructure	This aspect often well-addressed highlighted the importance of IT infrastructure in integration.
	Relational Connections and Compatibility	This aspect was less frequently reported, indicating potential gaps in understanding relational dynamics and compatibility within hospital settings.
Individual Characteristics	Need	The need for integration was unanimously recognised in all studies, emphasising its widespread acknowledgement.
	Capability, Opportunity, and Motivation	Few studies reported on these individual characteristics, indicating variable attention to them [42, 43, 50, 56, 58–61].
Implementation Process	Teaming, Assessing Context, and Doing	This aspect was reported inconsistently, indicating an area of focus for future research.
	Planning and Tailoring Strategies	Less frequently addressed, suggesting an area for improvement in future implementations.
	Reflecting & Evaluating and Adapting	Often overlooked, this highlighting the need for focused attention on the evaluation and adaptation phases.

## Quality Assessment of Pre-Post Studies with No Control Group

The Quality Assessment of Pre-Post Studies with No Control Group criteria was used to assess the strengths and limitations of the studies, focusing on the methodology, data reliability, and validity of the conclusions drawn (Table 3) [40]. Quality assessment revealed that most studies had clearly defined objectives; however, there was a notable lack of consistency in terms of eligibility and selection criteria. It was often unclear how representative the participants were of the wider population, and many studies failed to report on the enrolment process of eligible participants.

**Table 3:** NHLBI Quality Assessment Tool for Before-After (Pre-Post) Studies with No Control Group [40]

	Included studies																						
Quality Assessment Tool for Before-After (Pre-Post) Studies with No Control Group Criteria	1 [41]	2 [54]	3 [42]	4 [43]	5 [59]	6 [44]	7 [58]	8 [56]	9 [48]	10 [45]	11 [49]	12 [57]	13 [63]	14 [60]	15 [61]	16 [55]	17 [46]	18 [62]	19 [50]	20 [51]	21 [52]	22 [53]	23 [47]
1. Was the study question or objective clearly stated?	✓	✓	✓	✓	✓	✓	✓	CNM	✓	✓	✓	✓	✓	✓	✓	✓	✓	✓	✓	✓	✓	✓	✓
2. Were eligibility/selection criteria clearly described?	CNM	CNM	✓	✓	✓	✓	✓	CNM	✓	✓	✓	✓	✓	✓	✓	✓	✓	✓	✓	✓	✓	✓	✓
3. Were the participants representative of the target population?	CND	CND	CND	CND	CND	CND	CND	CND	CND	CND	CND	NR	CND	CND	CND	CNM	NR	CND	CNM	CND	CNM	CND	CND
4. Were all eligible participants enrolled?	CND	CND	CND	CND	CND	✓	CND	CND	NR	NA	NR	NR	CND	✓	CND	CNM	CND	CND	CNM	CND	✓	✓	✓
5. Was the sample size sufficient?	CND	NR	✓	CND	CND	NR	NR	NR	NR	✓	CND	NR	CND	CND	CND	CNM	CND	CND	CNM	CND	CNM	CND	NR
6. Was the intervention clearly described and consistently delivered?	✓	✓	✓	✓	✓	✓	✓	✓	✓	✓	✓	✓	✓	✓	✓	✓	✓	✓	✓	✓	✓	✓	✓
7. Were outcome measures valid, reliable, and consistently assessed?	✓	NR	✓	NR	CND	CND	✓	CND	✓	CNM	✓	✓	CND	✓	✓	✓	✓	✓	✓	✓	✓	✓	✓
8. Were outcome assessors blinded to exposures/interventions?	NR	NR	NR	NR	NR	CND	NR	NR	CND	NA	CND	NR	CND	CND	CND	CNM	NR	CND	CNM	NA	NR	CND	NR
9. Was loss to follow-up 20% or less, and accounted for in the analysis?	NR	NR	NR	NR	NR	NR	NR	NR	NR	CND	CND	NR	CND	CND	CND	CNM	CND	CND	CNM	CND	CND	CND	NR
10. Did the statistical methods examine pre-post changes and provide p values?	NR	NR	✓	✓	✓	✓	✓	NR	✓	✓	✓	✓	CND	✓	✓	✓	✓	✓	✓	✓	✓	✓	✓
11. Was an interrupted time series design used for outcome measures?	CNM	CNM	✓	CNM	✓	CNM	✓	CNM	CND	CNM	✓	CNM	✓	✓	✓	CNM	CNM	✓	CNM	✓	✓	CNM	CNM
12. Did the analysis account for individual-level data when conducting group-level analysis?	NR	NR	NR	NR	NR	NR	NR	NR	CND	CND	CND	NR	CND	✓	CND	CNM	NR	CND	CNM	CND	NR	CND	✓

Key:

• ✓—criteria met

• CNM—criteria not met fully

• NR—not reported

• CND—could not be determined

• NA—not applicable

Often studies did not report or determine the adequacy of their sample sizes, raising concerns regarding the statistical power and generalisability of their findings. Despite this, most studies consistently described and implemented interventions and used valid, reliable, and consistent measures for outcome assessments, even though a wide variety of outcome measures were used. Blinding of outcome assessors was not commonly reported or deemed applicable in these studies.

Many studies did not report or determine the loss to follow-up, nor did they provide specifics of their analysis methods. Additionally, some studies lacked adequate reporting of pre-post changes and associated P-values, and for some, this was not relevant due to the type of study design. The use of an interrupted time-series design was uncommon, which limited the ability to observe trends over time. Furthermore, reports on how individual-level data are incorporated into group-level analysis were often absent.

## Discussion

The purpose of this scoping review was to identify and evaluate studies investigating the effectiveness and implementation strategies of digital technologies integrated with EHR to improve medication-related outcomes and promote health equity among hospitalised adults. The review was guided by two objectives: first, to appraise studies that evaluate the effectiveness of EHR-digital technology in hospital medication management, specifically examining medication-related and health equity outcomes.; second, to identify and analyse the factors influencing the implementation of EHR-digital technology within complex hospital systems.

### Implications for Literature

Analysing 23 studies in 11 countries from 2008 to 2022, it was found that the studies used various research methodologies and investigated digital technologies such as CDSS, predictive analytics, real-time screening, and surveillance within EHR systems. These technologies demonstrated potential to reduce ADE, medication errors, medication discrepancies, potentially inappropriate medications, improve prescription accuracy, and optimise patient safety. Empirical support from recent meta-analyses and systematic reviews supports the efficacy of electronic prescribing, computerised order entry, and CDSS interventions [64–66].

The outcome measures were multifaceted and included metrics such as the rate of alert overrides, continuation of high-risk medication prescriptions, acceptance rates of automated recommendations, and the positive predictive value of triggers for the identification of ADE. The lack of a consistent definition of medication harm led to various approaches in its measurement and reporting [67, 68]. This inconsistency highlights the need for standardised definitions when developing EHR-digital technologies for medication management to enable more accurate reporting and comparison across studies.

Although these studies highlighted the role of EHR-digital technologies in improving medication safety, they also indicated the need for further research and system refinements. Issues such as alert fatigue from clinically irrelevant alerts, workflow assimilation, unstructured data, data quality, stakeholder approval, and the need for continuous staff training and support were identified [69–71]. Addressing these challenges is crucial to realise the full potential of these technologies. A comprehensive understanding and evaluation of these technologies in clinical settings is essential, focusing on factors that affect the trust of healthcare professionals, such as system design in alignment with clinical workflows, the clinical relevance of alerts, and adaptability to various clinical settings [72].

One of the key findings from this scoping review is the significant gap in focus on health equity. Despite the inclusion of 23 studies examining EHR-digital technologies and their effectiveness in medication management, none of these papers explored health and social determinants at any phase of the EHR-digital technology development, implementation, and evaluation. This represents a critical knowledge gap regarding the impact of these technologies on health equity. To address this, future studies must prioritise strategies and policies to leverage digital health solutions for equitable health outcomes in diverse patient populations [73].

### Effectiveness of EHR-digital Technologies in Medication Management

The integration of digital technologies within EHR systems is a much-needed area of research, with CDSS, real-time surveillance, predictive analytics, and AI driving the advancement and transformation of medication management. This review of 23 studies demonstrates the adaptability of EHR-digital technologies to various research settings, objectives and highlights the complexity of comparing and synthesising findings.

Effective digital health technologies are contingent upon careful consideration of design, integration, and iterative improvements, informed by clinical feedback and ongoing technology advancements. A tailored and customised approach is necessary, as a single solution may not be universally applicable in different clinical settings.

The complexity of EHR-digital technologies is further complicated by the integration of internal and third-party developed CDSS [74, 75]. This poses challenges for seamless integration, as digital tools may not always align perfectly with the native functionalities of EHR systems [74, 75]. Given the persistent nature of medication harm, the development, implementation and evaluation of CDSS has become a moving target, adding layers of complexity for healthcare providers that want to keep up with the growing demands, evolving standards and obtaining the most benefits for patients from these digital tools [74, 75].

A persistent issue is the lack of standardisation in alert mechanisms, leading to inconsistencies in the usability and clinical relevance of alerts across different systems. Although some systems seamlessly integrate into healthcare professional decision-making processes, others contribute to alert fatigue, which can cause healthcare providers to overlook critical alerts [76]. Alert algorithms need to be refined to ensure they are sensitive and specific, minimising the noise of irrelevant alerts while maximising the signal of clinically relevant information. Effective integration and implementation of digital alert systems are crucial, requiring seamless interoperability, end-user training, and a culture of safety that supports technology-enhanced clinical judgment [1, 4, 8].

The potential of digital alert systems to predict and prevent medication errors represents a significant technological advance, however, improving prediction accuracy requires an iterative design process. This process should incorporate clinical feedback, ongoing performance evaluation, and the application of AI and ML algorithms capable of evolving with experience [77].

Standardisation is also essential to define medication harm, harm classification and measurement tools to enable accurate and timely identification, monitoring, and evaluation, thus improving the reliability of integration of healthcare technology [78].

The integration of AI into EHR has the potential to revolutionise medication management using advanced algorithms and machine learning to process complex patient data. These tools can detect drug interactions, assess medication safety, and offer personalised clinician recommendations [79]. However, the application of AI in healthcare needs to mitigate algorithmic bias, which can exacerbate health inequities [80, 81]. The use of diverse datasets that incorporate digital and social health determinants is vital to tackle such biases [80, 81]. Moreover, the inclusion of standardised disability data is essential in the development and validation of AI technologies, ensuring equitable health outcomes [80, 81].

### Implications for Health Equity

The potential of digital healthcare technologies to enhance health equity is significant yet underexplored, with the literature exhibiting a critical research gap. A recent review highlighted a study conducted in an intensive care unit (ICU), which used a commercial EHR system to investigate the relationship between patient-identified race and the frequency of CDSS alert overrides, with a focus on drug-drug interaction alerts [74]. This study found a 1% higher rate of CDSS alert overrides for African-American patients compared to Caucasian patients (82.27% vs. 81.30%) [74]. This finding, although not fully explained, suggests that patient demographics may influence alert response behaviours, potentially influenced by factors such as polypharmacy or complex infusion regimens common in ICUs [74].

To achieve equity through digital innovations, intentional and informed efforts are needed. The Digital Health Equity Framework (DHEF) and the Health Equity Impact Assessment (HEIA) provide models for designing and evaluating digital health interventions with an equity focus [82, 83]. A study on a digital equity dashboard in an emergency department exemplifies how EHR data can inform interventions to reduce care disparities by analysing demographic and clinical variables [84].

Similarly, a digital equity dashboard for medication harm within EHR systems could highlight inequities across age, race, ethnicity, language, sexual orientation, gender identity, disability, and socioeconomic status. Engaging communities disproportionately affected by health inequities is crucial, as is incorporating their perspectives into research and monitoring digital technology performance to mitigate bias [33, 85, 86].

Interdisciplinary collaboration is essential to create digital health solutions that are not only technically robust, but also socially responsible and culturally sensitive. Health policy should address ethical considerations and allocate resources to technologies aimed at reducing health disparities, ensuring equitable access to technological advances [73].

Future research must use methodologies that assess the impact of digital technologies on diverse populations, including participatory design and longitudinal studies [87]. An international perspective is vital to share knowledge and strategies to address health inequities globally [88]. Ultimately, determining whether EHR-integrated digital tools can mitigate or exacerbate inequities in hospitalised patients is crucial to achieving the promise of technology in promoting equitable health outcomes.

### Factors Influencing Implementation in Hospital Systems

Implementing EHR-digital technology in hospitals is complex and involves more than just the technology itself. Institutional readiness, staff proficiency, organisational culture, and commitment to digital initiatives are crucial to success. Implementation requires a holistic strategy that integrates training, support, and alignment with clinical objectives.

The CFIR reveals strengths and areas for improvement. While research often highlighted the novelty of technology [41–46, 48–51, 54–60, 62, 63], there was a deficit in evidence-based practice support, questioning the reliability and generalisability of interventions [46, 48, 51, 54, 55, 60, 63].

The advantages and adaptability of digital technologies were well documented [41–45, 49, 51, 54–60, 63], however, EHR-digital technology design and cost considerations, which are critical for user adoption and financial viability, were not adequately addressed. It is crucial to evaluate the cost effectiveness of these systems using extensive research methods, as the economic aspects of incorporating digital technologies into EHR systems can be substantial and diverse, potentially impeding further innovation.

Studies typically focused on IT infrastructure and neglected the influence of factors such as critical incidents, local attitudes, policies, relational dynamics, and resources. Organisational culture, particularly in terms of equity and centeredness, whether it is recipient, deliverer, or learning-oriented, was rarely discussed [39, 41–63, 89].

Effective internal communication is vital for technology integration. However, external factors, such as policies and laws, which have a significant impact, were often overlooked. Additionally, the variability in individual capabilities and motivations indicates the need for more customised EHR-digital technology implementation approaches.

### Quality Assessment

The quality assessment of the studies indicates some significant strengths, such as clear research objectives that provide a solid foundation for the study focus and well-defined interventions and outcome metrics that enhance the reproducibility of the research and the credibility of the results. However, there are also some concerns, including potential selection bias due to ambiguous representativeness of study participants, and insufficient details on the inclusion of eligible participants and sample size calculation, which question the generalisability and applicability of the findings. Therefore, it is essential to develop improved reporting standards to address these gaps and strengthen the validity of future research efforts [90, 91].

### Challenges and Limitations in the Integration of EHR-Digital Technology

Integrating digital technologies into EHR systems is challenging, as highlighted in the included studies. Key challenges include alert fatigue, usability issues in the interface, and the complexities of integrating digital technologies into established clinical workflows. The exploration of results is limited by methodological shortcomings, such as the overestimation of positive predictive values due to inconsistent definitions of medication harm [43, 48, 49, 57, 58] and flawed study designs. To overcome these challenges, standardised research frameworks are needed, with a long-term evaluation of data security, patient and health equity outcomes [89].

To improve the reliability and comparability of the results, larger samples, broader data sources, and control groups are needed [43, 48, 49, 57, 58]. Staff adjustments, cost, workflows, ergonomic design, and customisation of alerts based on individual patient data are all essential components of adapting digital technologies in clinical practice [47, 53]. In addition, data quality concerns have been raised due to the retrospective nature of some studies. The challenges in interpreting unstructured data and integrating them with clinical laboratory and pharmacy information reflect the complexity of creating effective digital health technologies [44, 63]. Using structured clinical and patient data, as well as improving generalisability by including more hospitals, are needed [50, 62].

### Implications for Practice and Future Research

To maximise the benefits of EHR-digital technologies in medication management, a collaborative effort is needed to address implementation challenges, advance health equity, and create patient-centred solutions. Future research must assess the potential of emerging technologies, such as AL and ML, to improve medication management decision making as digital healthcare evolves.

Attention must be paid to cost–benefit analyses, system reliability, safety, comprehensive testing, cybersecurity, privacy, and ethical considerations [92]. Security and privacy in EHR systems, as targets of cyber threats, require rigorous surveillance and robust protective measures [93]. Technologies must align with clinical workflows and address patient requirements, with a focus on their long-term impacts on patient outcomes and health equity.

Usability and training are vital to effective adoption of the EHR system. Ongoing support and training for healthcare professionals, potentially through clinical simulations, can optimise system usage. The challenges of interoperability and data integration call for improved standards to facilitate seamless communication and data sharing among digital health platforms.

Patient-centred design is crucial, and integrating patient feedback into the development and implementation of technologies can lead to more responsive digital health solutions. Codesign methods prove valuable in aligning these solutions with patient needs.

### Limitations of This Review

To capture all relevant studies, our methodology used defined inclusion criteria across four databases, complemented by an electronic platform for study selection and review. Despite these efforts, the possibility of overlooking significant studies remains, in part due to the single-reviewer approach, which could introduce selection bias.

Examining peer-reviewed studies ensures quality, but excludes grey literature, which can offer valuable, although less scrutinised, insights to practice. Limiting our review to hospitalised adult patients and sources in English may have narrowed our perspective; however, this is the future context in which our work will be conducted.

The NHLBI Quality Assessment Tool was used to assess methodological quality and bias risk, a choice appropriate for our study design. However, this tool's focus does not fully accommodate studies that were not structured as pre-post comparisons or lacked control groups. Therefore, our results should be interpreted with caution, considering the methodological limitations and the specific focus of the assessment tool.

## Conclusion

This scoping review evaluated the effectiveness of digital technologies and their implementation strategies with EHRs, focussing on medications-related and health equity outcomes for hospitalised adults. Our findings suggest that these technologies hold considerable promise for improving patient safety, minimising medication harm, and enhancing health equity. However, the effectiveness of the implementation strategies varied, highlighting the need to address several challenges. Key challenges identified include alert fatigue, workflow integration, cost, data quality, and interoperability issues. Addressing these challenges is essential to fully realise the benefits of EHR-integrated digital technologies. Additionally, a significant gap in the literature is the lack of studies addressing health and social determinants during the development, implementation, and evaluation of these technologies. Future research should prioritise these areas to ensure that digital health solutions promote equitable outcomes. There is also a critical need to understand whether EHR-integrated digital technologies can reduce or exacerbate health inequities among hospitalised patients. By advancing our understanding and application of EHR-integrated digital technologies, we can move closer to achieving equitable and optimal medication-related outcomes for all hospitalised patients.

## Supplementary Information

Below is the link to the electronic supplementary material.

Supplementary file1 (XLSX 107 KB)

Supplementary file2 (DOCX 70 KB)

## Data Availability

No datasets were generated or analysed during the current study.

## Abbreviations

AI: Artificial Intelligence
ACE: Angiotensin-converting enzyme
ADE: Adverse Drug Event
ADR: Adverse Drug Reaction
CDSS: Clinical Decision Support System
CFIR: Consolidated Framework for Implementation Research
CPOE: Computerised Physician Order Entry
DAAS: Drug Allergy Alert Systems
DRP: Drug-Related Problem
EHR: Electronic Health Record
eMMS: Electronic Medication Management System
JBI: Joanna Briggs Institute
IOM: Institute Of Medicine
ME: Medication Error
ML: Machine Learning
PIM: Potentially Inappropriate Medication
PPV: Positive Predictive Value
PRISMA-Scr: Preferred Reporting Items for Systematic reviews and Meta-analyses extension for scoping reviews
WHO: World Health Organisation
