# Supplementary material for: Evaluating EHR-Integrated Digital Technologies for Medication-Related Outcomes and Health Equity in Hospitalised Adults: A Scoping Review
Source: J Med Syst. 2024 Aug 23;48(1):79. doi: 10.1007/s10916-024-02097-5 (PMC11341601; doi:10.1007/s10916-024-02097-5)
Supplement: Supplementary file 2 — Supplementary file2 (DOCX 70 KB) [file 10916_2024_2097_MOESM2_ESM.docx]

The Evaluation of EHR-Digital Technology Use in Enhancing Medication Management for Hospitalised Adults: A Scoping Review

## Journal of Medical Systems

Sreyon Murthi^1^ Dr Nataly Martini^1^ Dr Nazanin Falconer^2^Associate Professor Dr. Shane Scahill^1^

1. University of Auckland, School of Pharmacy, Auckland, New Zealand
2. University of Queensland, School of Pharmacy, Brisbane, Australia

ORCID:

Sreyon Murthi - 0000-0002-7419-7083

Dr. Nataly Martini – 0000-0002-9900-8883

Dr. Nazanin Falconer –

Dr. Shane Scahill - 0000-0001-5350-696X

Corresponding Author:

Sreyon Murthi

The University of Auckland

School of Pharmacy, Faculty of Medical & Health Sciences

Auckland, New Zealand

Email: [sreyon.murthi@auckland.ac.nz](mailto:sreyon.murthi@auckland.ac.nz)

Consolidated Framework for Implementation Research (CFIR) domains for the included studies [47]

|  | | Included studies | | | | | | | | | | | | | | | | | | | | | | |
| --- | --- | --- | --- | --- | --- | --- | --- | --- | --- | --- | --- | --- | --- | --- | --- | --- | --- | --- | --- | --- | --- | --- | --- | --- |
| CFIR main domain | Subdomain | 1  [49] | 2  [69] | 3  [50] | 4  [51] | 5  [67] | 6  [52] | 7  [66] | 8  [65] | 9  [60] | 10  [53] | 11  [61] | 12  [71] | 13  [54] | 14  [55] | 15  [56] | 16  [68] | 17  [57] | 18  [58] | 19  [62] | 20  [63] | 21  [64] | 22  [70] | 23  [59] |
| Intervention |  | | | | | | | | | | | | | | | | | | | | | | | |
|  | Innovation Source |  | ✓ | ✓ | ✓ | ✓ |  | ✓ | ✓ | ✓ | ✓ | ✓ | ✓ | ✓ | ✓ | ✓ | ✓ | ✓ | ✓ | ✓ | ✓ | ✓ | ✓ | ✓ |
|  | Evidence-base |  | ✓ | ✓ |  |  |  |  |  |  | ✓ | ✓ |  | ✓ |  |  | ✓ |  | ✓ |  | ✓ |  | ✓ |  |
|  | Relative Advantage |  | ✓ | ✓ | ✓ | ✓ | ✓ | ✓ | ✓ |  |  |  |  | ✓ | ✓ | ✓ | ✓ | ✓ | ✓ |  | ✓ | ✓ | ✓ | ✓ |
|  | Adaptability |  | ✓ | ✓ |  | ✓ |  | ✓ |  |  | ✓ |  |  | ✓ |  |  | ✓ |  | ✓ | ✓ | ✓ | ✓ | ✓ | ✓ |
|  | Trialability | ✓ | ✓ | ✓ | ✓ | ✓ | ✓ | ✓ | ✓ | ✓ | ✓ | ✓ | ✓ | ✓ | ✓ | ✓ | ✓ | ✓ | ✓ | ✓ | ✓ | ✓ | ✓ | ✓ |
|  | Complexity | ✓ | ✓ | ✓ | ✓ | ✓ | ✓ | ✓ | ✓ | ✓ | ✓ | ✓ | ✓ | ✓ | ✓ | ✓ | ✓ | ✓ | ✓ | ✓ | ✓ | ✓ | ✓ | ✓ |
|  | Design |  | ✓ |  | ✓ |  |  |  | ✓ |  |  |  |  |  |  |  |  |  | ✓ |  | ✓ |  | ✓ | ✓ |
|  | Cost |  |  |  |  |  |  |  |  |  |  |  |  |  |  |  |  |  |  |  |  |  |  |  |
| Outer setting |  | | | | | | | | | | | | | | | | | | | | | | | |
|  | Critical incidents |  |  |  |  |  |  |  |  |  |  |  |  |  |  |  |  |  |  |  |  |  |  |  |
|  | Local attitudes |  |  |  |  |  |  |  |  |  |  |  |  |  |  |  |  |  |  |  |  |  |  |  |
|  | Local conditions |  |  |  |  |  |  |  |  |  |  |  |  |  |  |  |  |  |  |  |  |  |  |  |
|  | Partnerships & connections |  | ✓ |  |  |  |  |  |  |  |  |  |  |  |  |  |  |  |  |  |  |  |  |  |
|  | Policies & laws |  |  |  |  |  |  |  |  |  |  |  |  |  |  |  |  |  |  |  |  |  |  |  |
|  | Financing |  |  |  |  |  |  |  |  |  |  |  |  |  |  |  |  |  |  |  |  |  |  |  |
|  | External pressure |  |  |  |  |  |  |  |  |  |  |  |  |  |  |  |  |  |  |  |  |  |  |  |
|  | - Societal pressure |  |  |  |  |  |  |  |  |  |  |  |  |  |  |  |  |  |  |  |  |  |  |  |
|  | - Market pressure |  |  |  |  |  |  |  |  |  |  |  |  |  |  |  |  |  |  |  |  |  |  |  |
|  | - Performance-measurement pressure |  |  |  |  |  |  |  |  |  |  |  |  |  |  |  |  |  |  |  |  |  |  |  |
| Inner setting |  | | | | | | | | | | | | | | | | | | | | | | | |
|  | Structural Characteristics |  |  |  |  |  |  |  |  |  |  |  |  |  |  |  |  |  |  |  |  |  |  |  |
|  | - Physical infrastructure |  |  |  |  |  |  |  |  |  |  |  |  |  |  |  |  |  |  |  |  |  |  |  |
|  | - Information technology infrastructure | ✓ | ✓ | ✓ | ✓ | ✓ | ✓ | ✓ | ✓ | ✓ | ✓ | ✓ | ✓ | ✓ | ✓ | ✓ | ✓ | ✓ | ✓ | ✓ | ✓ | ✓ | ✓ | ✓ |
|  | - Work infrastructure |  |  |  |  |  |  |  |  |  |  |  |  |  |  |  |  |  |  |  |  |  |  |  |
|  | Relational connections |  |  |  |  |  |  | ✓ |  |  |  |  |  |  |  |  | ✓ |  | ✓ |  |  |  |  |  |
|  | Communications |  |  |  |  |  |  | ✓ |  |  |  |  |  |  |  |  |  |  |  |  |  |  |  |  |
|  | Culture |  |  |  |  |  |  |  |  |  |  |  |  |  |  |  |  |  |  |  |  |  |  |  |
|  | - Human equality-centredness |  |  |  |  |  |  |  |  |  |  |  |  |  |  |  |  |  |  |  |  |  |  |  |
|  | - Recipient-centredness |  |  |  |  |  |  |  |  |  |  |  |  |  |  |  |  |  |  |  |  |  |  |  |
|  | - Deliverer-centredness |  |  |  |  |  |  |  |  |  |  |  |  |  |  |  |  |  |  |  |  |  |  |  |
|  | - Learning-centredness |  |  |  |  |  |  |  |  |  |  |  |  |  |  |  |  |  |  |  |  |  |  |  |
|  | Tension for change |  |  | ✓ |  | ✓ |  | ✓ |  |  |  |  |  |  |  |  |  |  |  |  |  |  |  |  |
|  | Compatibility | ✓ | ✓ | ✓ | ✓ | ✓ | ✓ | ✓ | ✓ | ✓ | ✓ | ✓ | ✓ | ✓ | ✓ | ✓ | ✓ | ✓ | ✓ | ✓ | ✓ | ✓ | ✓ | ✓ |
|  | Relative priority |  |  | ✓ |  |  |  |  |  |  |  |  |  |  |  |  |  |  |  |  |  |  |  |  |
|  | Incentive systems |  |  |  |  |  |  |  |  |  |  |  |  |  |  |  |  |  |  |  |  |  |  |  |
|  | Mission alignment |  |  |  |  |  |  |  |  |  |  |  |  |  |  |  |  |  |  |  |  |  |  |  |
|  | Available resources |  |  |  |  |  |  |  |  |  |  |  |  |  |  |  |  |  |  |  |  |  |  |  |
|  | - Funding |  |  |  |  |  |  |  |  |  |  |  |  |  |  |  |  |  |  |  |  |  |  |  |
|  | - Space |  |  |  |  |  |  |  |  |  |  |  |  |  |  |  |  |  |  |  |  |  |  |  |
|  | - Materials & Equipment |  |  |  |  |  |  |  |  |  |  |  |  |  |  |  |  |  |  |  |  |  |  |  |
|  | Access to knowledge and information |  | ✓ | ✓ |  | ✓ |  | ✓ | ✓ |  |  |  |  |  |  |  |  |  |  | ✓ |  |  |  |  |
| Individual characteristics |  | | | | | | | | | | | | | | | | | | | | | | | |
|  | High-level Leaders |  |  |  |  |  |  |  |  |  |  |  |  |  |  |  |  |  |  |  |  |  |  |  |
|  | Mid-level Leaders |  |  |  |  |  |  |  |  |  |  |  |  |  |  |  |  |  |  |  |  |  |  |  |
|  | Opinion Leaders |  |  |  |  |  |  |  |  |  |  |  |  |  |  |  |  |  |  |  |  |  |  |  |
|  | Implementation Facilitators |  |  |  |  |  |  |  |  |  |  |  |  |  |  |  |  |  |  |  |  |  |  |  |
|  | Implementation Leads |  |  |  |  |  |  |  |  |  |  |  |  |  |  |  |  |  |  |  |  |  |  |  |
|  | Implementation Team Members |  |  |  |  |  |  |  |  |  |  |  |  |  |  |  |  |  |  |  |  |  |  | ✓ |
|  | Other Implementation Support |  |  |  |  |  |  |  |  |  |  |  |  |  |  |  |  |  |  |  |  |  |  |  |
|  | Innovation Deliverers |  |  |  |  |  |  |  |  |  |  |  |  |  |  |  |  |  |  |  |  |  |  |  |
|  | Innovation Recipients |  |  |  |  |  |  |  |  |  |  |  |  |  |  |  |  |  |  |  |  |  |  |  |
|  | Characteristics |  |  |  |  |  |  |  |  |  |  |  |  |  |  |  |  |  |  |  |  |  |  |  |
|  | - Need | ✓ | ✓ | ✓ | ✓ | ✓ | ✓ | ✓ | ✓ | ✓ | ✓ | ✓ | ✓ | ✓ | ✓ | ✓ | ✓ | ✓ | ✓ | ✓ | ✓ | ✓ | ✓ | ✓ |
|  | - Capability |  |  | ✓ | ✓ | ✓ |  | ✓ | ✓ |  |  |  |  |  | ✓ | ✓ |  |  |  | ✓ |  |  |  |  |
|  | - Opportunity |  |  | ✓ | ✓ | ✓ |  | ✓ | ✓ |  |  |  |  |  |  |  |  |  |  | ✓ |  |  |  |  |
|  | - Motivation |  |  |  |  |  |  |  | ✓ |  |  |  |  |  |  |  |  |  |  | ✓ |  |  |  |  |
| Implementation process |  | | | | | | | | | | | | | | | | | | | | | | | |
|  | Teaming |  |  | ✓ |  |  |  | ✓ |  |  |  |  | ✓ |  | ✓ | ✓ |  |  |  | ✓ |  |  |  | ✓ |
|  | Assessing Needs |  |  |  |  |  |  |  |  |  |  |  |  | ✓ |  |  |  |  |  | ✓ |  |  |  |  |
|  | - Innovation Deliverers |  |  |  |  |  |  |  |  |  |  |  |  |  |  |  |  |  |  |  |  |  |  |  |
|  | - Innovation Recipients |  | ✓ |  |  | ✓ |  | ✓ |  |  |  |  |  |  |  |  |  |  |  | ✓ |  |  |  |  |
|  | Assessing Context |  |  | ✓ |  | ✓ |  | ✓ |  |  |  | ✓ | ✓ |  |  |  |  |  |  | ✓ |  |  |  |  |
|  | Planning |  |  |  |  | ✓ |  |  |  |  |  |  | ✓ |  | ✓ | ✓ |  |  |  |  |  |  |  |  |
|  | Tailoring Strategies |  |  |  |  |  |  | ✓ |  |  |  |  | ✓ |  | ✓ | ✓ |  |  |  |  |  |  |  |  |
|  | Engaging |  |  |  |  |  |  |  |  |  |  |  |  |  |  |  |  |  |  | ✓ |  |  |  |  |
|  | - Innovation Deliverers |  |  |  |  |  |  |  |  |  |  |  |  |  |  |  |  |  |  |  |  |  |  |  |
|  | - Innovation Recipients |  |  |  |  |  |  |  |  |  |  |  |  | ✓ |  |  |  |  |  |  |  |  |  |  |
|  | Doing |  |  | ✓ |  |  |  | ✓ |  |  |  |  | ✓ |  | ✓ | ✓ |  |  |  | ✓ |  |  |  | ✓ |
|  | Reflecting & Evaluating |  |  |  |  |  |  |  |  |  |  |  |  |  |  |  |  |  |  |  |  |  |  |  |
|  | - Implementation |  |  |  |  |  |  |  |  |  |  |  |  |  |  |  |  |  |  |  |  |  |  |  |
|  | - Innovation | ✓ | ✓ | ✓ | ✓ | ✓ | ✓ | ✓ | ✓ | ✓ | ✓ | ✓ | ✓ | ✓ | ✓ | ✓ | ✓ | ✓ | ✓ | ✓ | ✓ | ✓ | ✓ | ✓ |
|  | Adapting |  |  |  |  | ✓ |  | ✓ |  |  | ✓ |  | ✓ |  |  |  |  |  |  | ✓ |  |  |  |  |

Adapted from Damschroder LJ, Aron DC, Keith RE, Kirsh SR, Alexander JA, Lowery JC. The updated Consolidated Framework for Implementation Research based on user feedback [47]

Key:

✓ - domain criteria met (either full or partial)

Blank cell - could not be determined, not reported or not applicable.

A full description of the CFIR domains and subdomains can be found on the following website: <https://cfirguide.org/constructs/> [47]
